# PacBio-LITS: a large-insert targeted sequencing method for characterization of human disease-associated chromosomal structural variations

**DOI:** 10.1186/s12864-015-1370-2

**Published:** 2015-03-19

**Authors:** Min Wang, Christine R Beck, Adam C English, Qingchang Meng, Christian Buhay, Yi Han, Harsha V Doddapaneni, Fuli Yu, Eric Boerwinkle, James R Lupski, Donna M Muzny, Richard A Gibbs

**Affiliations:** Human Genome Sequencing Center, Baylor College of Medicine, Houston, TX 77030 USA; Department of Molecular and Human Genetics, Baylor College of Medicine, Houston, TX 77030 USA; Human Genetics Center, University of Texas Health Science Center at Houston, Houston, TX 77030 USA

**Keywords:** Targeted sequencing, Single molecule sequencing, Complex genomic rearrangement

## Abstract

**Background:**

Generation of long (>5 Kb) DNA sequencing reads provides an approach for interrogation of complex regions in the human genome. Currently, large-insert whole genome sequencing (WGS) technologies from Pacific Biosciences (PacBio) enable analysis of chromosomal structural variations (SVs), but the cost to achieve the required sequence coverage across the entire human genome is high.

**Results:**

We developed a method (termed PacBio-LITS) that combines oligonucleotide-based DNA target-capture enrichment technologies with PacBio large-insert library preparation to facilitate SV studies at specific chromosomal regions. PacBio-LITS provides deep sequence coverage at the specified sites at substantially reduced cost compared with PacBio WGS. The efficacy of PacBio-LITS is illustrated by delineating the breakpoint junctions of low copy repeat (LCR)-associated complex structural rearrangements on chr17p11.2 in patients diagnosed with Potocki–Lupski syndrome (PTLS; MIM#610883). We successfully identified previously determined breakpoint junctions in three PTLS cases, and also were able to discover novel junctions in repetitive sequences, including LCR-mediated breakpoints. The new information has enabled us to propose mechanisms for formation of these structural variants.

**Conclusions:**

The new method leverages the cost efficiency of targeted capture-sequencing as well as the mappability and scaffolding capabilities of long sequencing reads generated by the PacBio platform. It is therefore suitable for studying complex SVs, especially those involving LCRs, inversions, and the generation of chimeric *Alu* elements at the breakpoints. Other genomic research applications, such as haplotype phasing and small insertion and deletion validation could also benefit from this technology.

**Electronic supplementary material:**

The online version of this article (doi:10.1186/s12864-015-1370-2) contains supplementary material, which is available to authorized users.

## Background

In the past decade, large-scale DNA sequencing efforts such as the 1000 Genomes Project and the human haplotype map (HapMap) project have provided unprecedented insights into the pattern of DNA sequence variation in the human genome [1-3]. Both single nucleotide variants and submicroscopic chromosomal structural variants (SVs), which were defined as variants ranging from ~1 Kb to 3 Mb in size and mainly including copy number variations, low copy repeats (LCRs; also known as segmental duplications), inversions and translocations [4], have been discovered and extensively studied. Comprehensive characterization of SVs has, however, proved challenging and is exacerbated by the observation that SVs are often associated with LCRs and highly repetitive genomic features such as long interspersed elements and short *Alu* repetitive elements. Long and accurate sequencing read lengths, combined with sufficient base coverage, can, in some instances, allow SVs to be spanned by the mapped sequence data and provide precise location and size information. Alternatively, paired-end sequencing of large insert clones can be applied. The earliest end-sequence profiling of bacterial artificial chromosomes led to successful mappings of selected locus specific structural rearrangements in cancer genomes [5], but has not been routinely implemented because the process is expensive and labor-intensive. Other methods, based upon hybridization-based microarray technologies (*e.g.* array Comparative Genomic Hybridization (aCGH) and single nucleotide polymorphism array) have enabled researchers to screen entire genomes for chromosomal copy number gains or losses [6-8], but such assays generally have limited resolution and largely depend on both probe design and prior knowledge of human genome architecture.

Next-generation sequencing (NGS) read-pair mapping [9,10] as well as other derived computational methods such as read depth-based analyses [11] have much improved SV detection and characterization. NGS allows simultaneous discovery of multiple classes of variants with breakpoint junction resolution at the single nucleotide level. Nevertheless, short sequencing read lengths (50–400 bp) generated by the current major NGS platforms (Roche/454/GS FLX, Illumina/HiSeq2000/2500, Thermo Fisher/Ion Torrent) pose a significant challenge for data analyses due to the considerable read-mapping ambiguity in genomic regions containing repeats. Moreover, studies of chromosomal SVs associated with genomic disorders have provided evidence for further complexity than anticipated in both the formation [12] and the end products of rearrangement [13].

The PacBio RSII system is designed to perform single molecule, real-time sequencing [14], which is distinct from the “clonal amplification”-based sequencing conducted by other NGS platforms. The platform is capable of producing long sequencing reads (>20 Kb maximum read length) and is able to span repetitive sequences and breakpoint junctions of SVs. These unique properties have enabled solutions to some previously intractable biological problems, such as mapping of methylated bases in pathogenic microbes [15] and sequencing of disease-associated trinucleotide repeats [16]. While large-insert WGS can be conducted on the PacBio platform, the cost to achieve relatively deep coverage across the entire human genome for routine structural variation analysis is high.

To take advantage of the PacBio long reads and reduce costs, we developed PacBio-LITS, a large-insert targeted capture-sequencing method. PacBio-LITS leverages the cost efficiency of targeted capture-sequencing as well as the mappability and scaffolding capabilities of long sequencing reads generated by the PacBio platform. Here, we demonstrate the utility of the new method in a complex diagnostic scenario - determining LCR-mediated breakpoint junction sequences in non-recurrent duplications leading to a genomic disorder.

## Results and discussion

### PacBio-LITS overview

**Figure 1:**
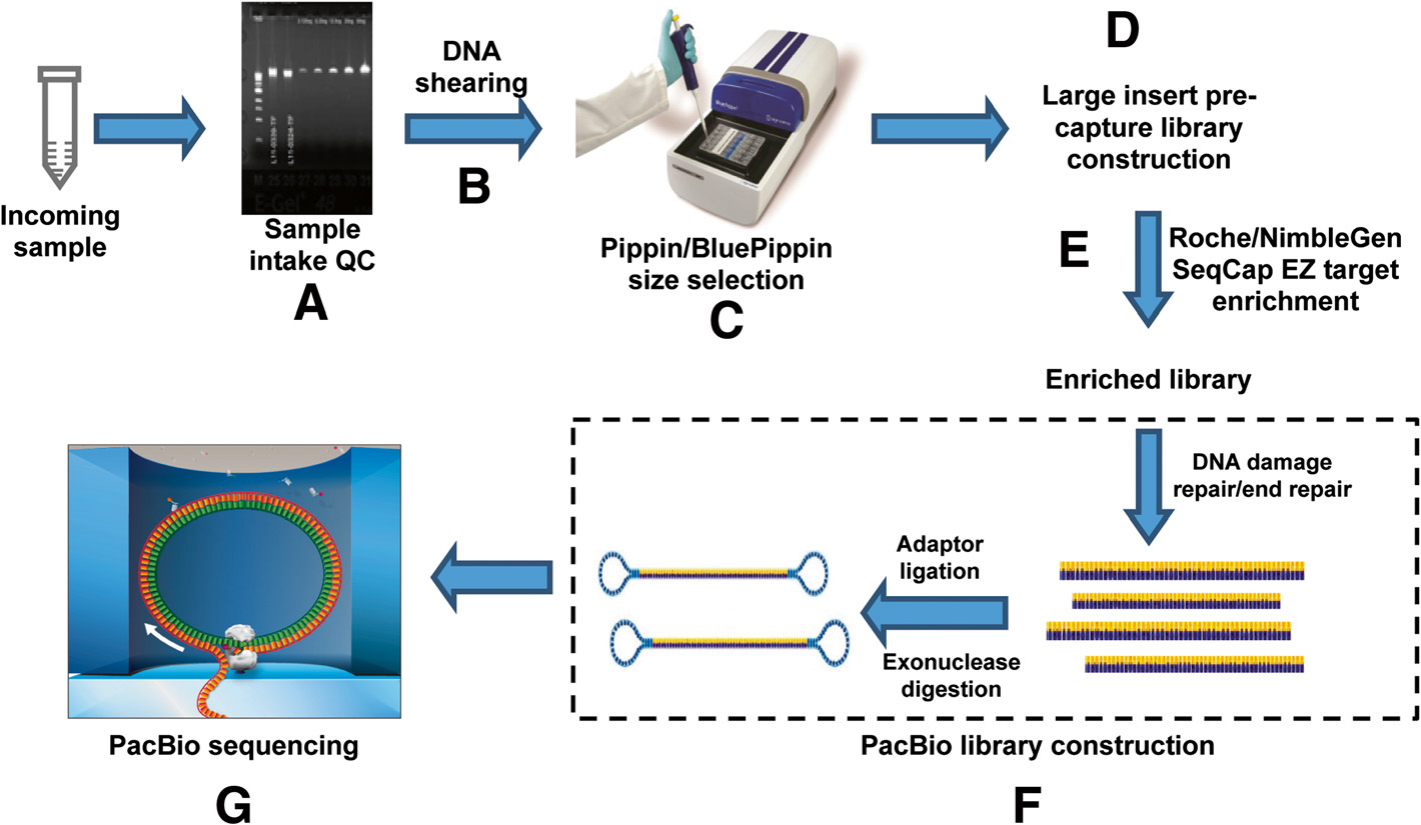
**Workflow of the PacBio-LITS method.** **A**. sample intake QC; **B**. DNA fragmentation; **C**. target size selection using Pippin/BluePippin; **D**. construction of pre-capture library using size-selected DNA fragments; **E**. Roche/NimbleGen SeqCap EZ solution-based target enrichment using custom probes; **F**. construction of PacBio library using post-capture product and G. PacBio long read-length sequencing.

The new method consists of two major steps: 1) large-insert capture library preparation, and 2) PacBio library preparation using the captured product as template (Figure 1). The sample intake QC step involves analysis by agarose gel electrophoresis to determine DNA integrity (*e.g.* intact or degraded) and measuring the sample concentration by PicoGreen dsDNA assay or Qubit fluorometric quantitation. DNA fragmentation is achieved by using Covaris Focused-ultrasonicator or a g-TUBE apparatus. Selection for the targeted insert size is performed using Sage Science’s Pippin (for 1 Kb insert) or BluePippin (for >1 Kb insert) platform under specific running parameters. The size-selected DNA fragments then undergo a pre-capture library preparation process that is similar to the standard Illumina paired-end library construction involving end repair, 3′-adenylation, adaptor ligation and ligation-mediated PCR (LM-PCR). Target enrichment of the pre-capture library DNA follows the Roche/NimbleGen liquid hybridization protocol using specific solution probes (SeqCap probes). The post-capture product serves as the input DNA for PacBio large-insert library preparation. The final product, a large insert capture library with PacBio SMRT bell adaptors ligated to both ends of the inserts, is loaded onto the PacBio platform for long read-length sequencing. A complete protocol for 6 Kb insert PacBio capture library constructions is appended (see Additional file 1: BCM-HGSC PacBio-LITS Protocol).

### Critical considerations for the experimental conditions

Long-range PCR coupled with PacBio amplicon sequencing has recently been utilized to elucidate recurrent somatic SVs in cancer samples [17]. However, the method generally requires careful design of a large set of tiling primers and is limited both by the size of breakpoint region and the ability to identify and amplify junctions that can be complex in nature. While long-read sequencing of target-capture libraries (250 bp - 2 Kb, no size selection) using the PacBio platform has proved successful for discovering novel single nucleotide variants in regions where probe design is difficult [18], targeted sequencing of large-insert (≥1 Kb) libraries for SV characterization has not been reported. To generate target-enriched, long sequencing reads from the PacBio platform for SV study, construction of high-quality, size-selected large-insert capture libraries is required. We determined that the following experimental conditions demand particular attention to achieve this (see Table 1).

**Table 1:** **Optimized conditions for PacBio-LITS library preparation**

**Experimental conditions**	**Library insert sizes**			
	**1 Kb**	**4 Kb**	**6 Kb**	**7-8 Kb**
**Input DNA**	500 ng	1 μg	1.5 μg	2 μg
**Shearing method**	Covaris	Covaris or g-TUBE	g-TUBE	g-TUBE
**Shearing condition**	1 Kb shearing with microTube	Covaris: 5 Kb shearing with miniTube o*r* g-TUBE: 12000 rpm, 45 sec.	7000 rpm, 3 min.	4800 rpm, 8 min.
**Size selection method**	Pippin	Blue Pippin	Blue Pippin	Blue Pippin
**Size selection condition**	Range mode: 900 bp-1.3 Kb	Range mode: 4 Kb-8 Kb	Range mode: 5 Kb-9 Kb	Range mode: 8 Kb-12 Kb
**DNA Polymerase**	Phusion HF Polymerase	TaKaRa LA *Taq* Polymerase (Hot-start)	TaKaRa LA *Taq* Polymerase (Hot-start)	TaKaRa LA *Taq* Polymerase (Hot-start)
**Pre-cap PCR cycle #**	6	8	8	10
**DNA amount into Capture**	1 μg	1 μg	2 μg	2 μg
**Post-cap PCR cycle #**	14	16	16	18
**Mean mapped subread length**	~700 bp	~1.8-2 Kb	~2-2.5 Kb	~2.5-3 Kb

#### Input DNA

Generally, as the insert size increases, the requisite amount of initial DNA required increases. For 1 Kb insert libraries, 500 ng of input DNA is adequate when following the PacBio-LITS protocol (Additional file 1: BCM-HGSC PacBio-LITS Protocol). For libraries with insert sizes up to 4 Kb, 1 μg of input DNA is generally sufficient. When the targeted library insert size is greater than 4 Kb, more template DNA should be used: we normally use 1.5 μg of DNA for 6 Kb insert library preparations and 2 μg of DNA for 8 Kb insert library preparations (Table 1). Note that the sample DNA quality will always affect the necessary amount of DNA. High-quality genomic DNA showing no sign of degradation has been considered a prerequisite for successful construction of large-insert (>10 Kb) PacBio libraries, and pulsed-field gel electrophoresis has been recommended by the manufacturer to carefully examine the integrity of the sample DNA (User Bulletin - Guidelines for Preparing 20 Kb SMRTbell™ Templates, pub date 2014-03-01). Although the PacBio-LITS method is theoretically more tolerant of compromised DNA quality (due to the inclusion of LM-PCR process), DNA degradation will still affect the library preparation (especially when the insert size is ≥4 Kb) and therefore more DNA may be required.

#### DNA fragmentation

To generate capture libraries with 1–4 Kb inserts, we recommend using Covaris’ Focused-ultrasonicator for DNA fragmentation. However, if the targeted insert size is ≥4 Kb, using Covaris’ g-TUBE products to shear the DNA is advised (Table 1). g-TUBE is a single-use device that shears genomic DNA into selected fragment sizes up to 20 kb. The only equipment needed for g-TUBE is a compatible bench-top centrifuge. The underlying principles for the two shearing methods are distinctively different: the Focused-ultrasonicator method employs the adaptive focused acoustics technology to mechanically shear the DNA using acoustic energy, whereas the g-TUBE method utilizes centrifugal force to move the sample through a precisely sized and manufactured orifice for fragmentation. For insert sizes smaller than 4 Kb, acoustic shearing exhibits more reliable performance. Usage of g-TUBEs for DNA fragmentation requires that the DNA be of high purity because the opening of g-TUBEs can be blocked by particles, resulting in inconsistent performance and occasionally considerable sample loss.

#### Size selection

DNA polymerases amplify small fragments more robustly than large fragments. To eliminate amplification bias, size-selection of the sheared DNA fragments is necessary and should be performed prior to LM-PCR amplification. This is especially relevant when preparing capture libraries with insert sizes greater than 4 Kb. Manual gel-extraction methods involving agarose gel electrophoresis can be used, but we have chosen Sage Science’s Pippin and BluePippin platforms to perform target size selection for improved accuracy and sample recovery (Table 1). Size selection can be performed after adaptor ligation, as in some standard library preparation protocols (*e.g.* the Illumina paired-end library preparation with gel extraction). For large-insert capture library construction, we have modified the procedure and performed size selection immediately preceding downstream enzymatic reactions. Thus, only fragments with the desired target size are subjected to enzymatic reactions. When operating Pippin and BluePippin instruments, we always select the “range mode” for size selection to retain DNA complexity.

#### Adaptor ligation

The optimal adaptor ligation in pre-capture library preparation is critical for efficient LM-PCR amplification. Currently, we employ Illumina “Y” adaptors for this purpose. Medium- or large-size targets (>1 Mb) normally demand one or more SMRT cells of sequencing to achieve sufficient base coverage. In such case non-barcoded “Y” adaptors can be used. However, for smaller targets (<1 Mb), utilization of molecular barcoding techniques allows multiplexing of samples and greater economy.

Additional considerations for multiplexing strategies are that the current PacBio P5 polymerase and C3 chemistry combination (P5*-*C3) results in a mean post-filtered read length of ~8 Kb (with a 180 movie time). For a large-insert PacBio-LITS library (*e.g.* ~6 Kb), the reads may not pass through the barcode sequence or may read through the sequence only once. With a high single-pass error rates (~15%), accurate decoding of individual reads requires multiple-pass (>3x) circular consensus sequencing of the barcodes, and is thus inefficient for these larger insert sizes. Utilization of longer barcodes may ameliorate this problem due to the ability to match a degenerate sequence more accurately.

#### DNA polymerase

Clean and robust LM-PCR amplification is critical for success of the new method. The DNA polymerase used should exhibit high processivity to generate robust and specific amplification of long inserts. In contrast, high fidelity of the DNA polymerase is not critical due to the sequencing error rate already present in reads using the PacBio platform. To prepare ≥4 Kb insert libraries, we use TaKaRa LA *Taq* Polymerase (Cat. # RR042, Clontech Lab. Inc.) for PCR amplification since it is more robust than other enzymes at generating long amplicons (Additional file 2: Figure S1). In addition to being a robust polymerase for the amplification of large genomic regions, this polymerase also has an error rate of approximately 8.7 x 10^−6^ per base incorporated, and therefore introduces far fewer errors than PacBio chemistry does. Additionally, point mutations close to breakpoint junctions are subjected to further examination during Sanger confirmation. We also limit the PCR cycle number to avoid non-specific amplification products.

#### Target enrichment

We currently employ the Roche/NimbleGen SeqCap EZ solution-based target enrichment method. Normally 1–2 μg of pre-capture library DNA is hybridized with solution probes. While it is important to minimize the post-capture LM-PCR cycle number, sufficient amplification cycles should be performed in order to generate >1 μg of capture products for the following PacBio library preparation. Due to the exonuclease digestion, PacBio large-insert library preparations usually give rise to low yields (~20-30%). In our tests, we normally conducted 14–18 cycles of post-capture PCR amplification (Table 1).

### PacBio-LITS pilot studies

**Figure 2:**
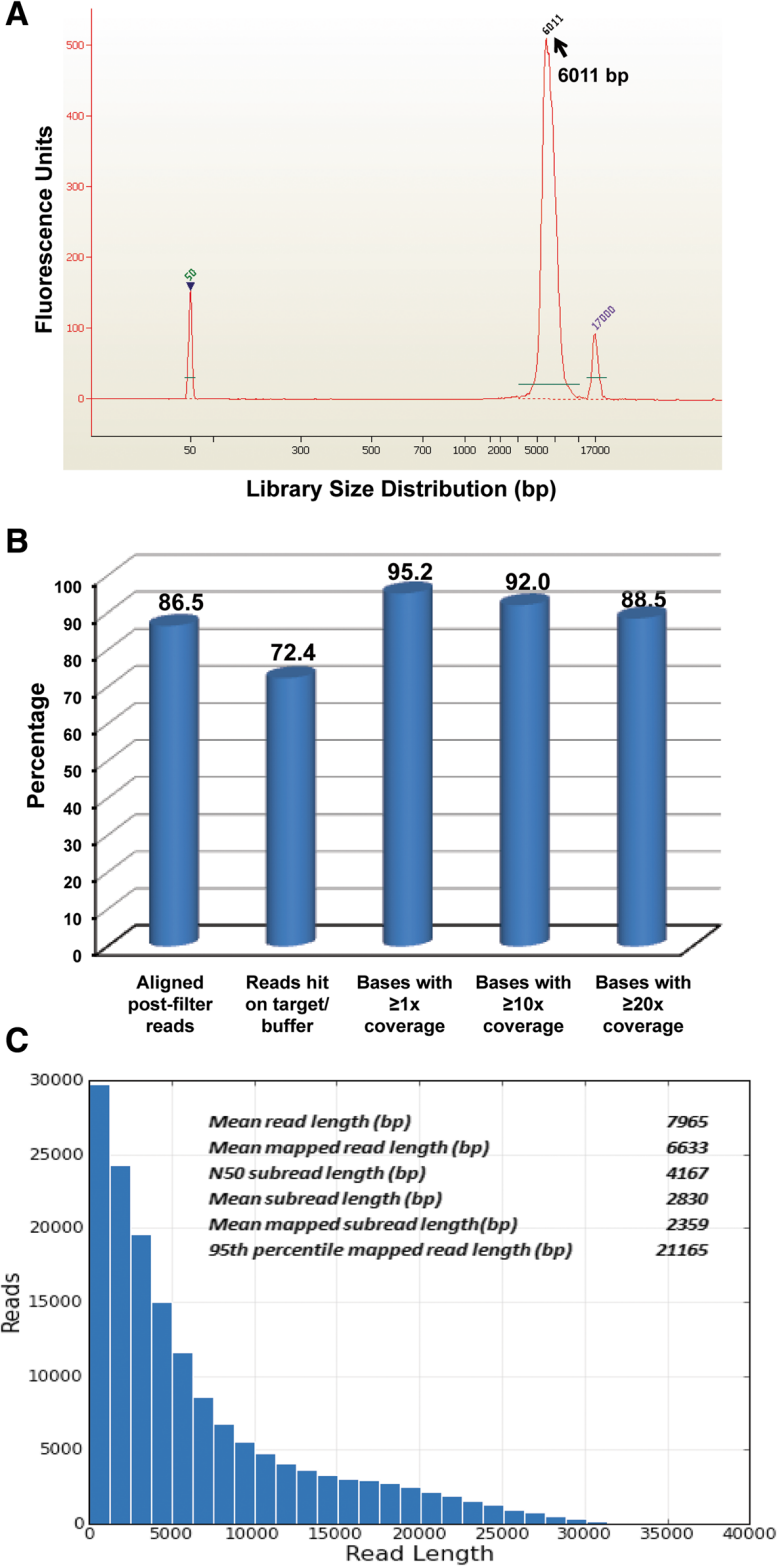
**PacBio sequencing of a 6 Kb insert capture library. A)** Post-capture library QC. A 6Kb insert capture library was constructed using the custom SMS/PTLS probe set and quality of the post-capture LM-PCR product was analyzed by Agilent 2100 Bioanalyzer. **B)** Target capture performance. Critical capture sequencing metrics (alignment rate, on-target/buffer rate and base coverage) were calculated after data analysis. **C)** Read length performance. Sequencing the 6 Kb insert library by PacBio C3/P5 chemistry with a 180-min movie generated a set of read length metrics for post-filter reads and subreads.

Multiple proof-of-concept tests were conducted using 1–6 Kb insert PacBio-LITS libraries (Table 2; the detailed results are shown in Additional file 3: Table S1). Two different probe sets were used in the sequence capture process: 1) SMS/PTLS, targeting a 7 Mb region (from 14.9 Mb to 21.9 Mb) on Chr.17p11.2 spanning the majority of duplication events leading to Potocki-Lupski syndrome (PTLS; MIM #610883) and deletion events causative of its reciprocal genomic disorder Smith-Magenis syndrome (SMS; MIM #182290) [19,20], and 2) human *Major Histocompatibility Complex* (*MHC)* from NimbleGen (Design Name: 110729_HG19_MHC_L2R_D03_EZ), which targets a 4.97 Mb region surrounding the human *MHC* loci. Genomic DNAs from HapMap NA12878, HGSC control sample HS1011 and the SMS patient sample BAB1123 were used in the library preparation. Five PacBio-LITS libraries were constructed by following the procedures described in Additional file 1: BCM-HGSC PacBio-LITS Protocol, including three with the SMS/PTLS probe set (1 Kb and 4 Kb insert from BAB1123 and a 6 Kb insert from NA12878) and two with the *MHC* probe set (4 Kb insert from both NA12878 and HS1011). These libraries were sequenced using different PacBio chemistries and movie times (Table 2; Additional file 3: Table S1). Capture and read length metrics were generated and exemplified by the NA12878 SMS/PTLS 6 Kb insert PacBio capture library in Figure 2. Approximately 87% of the post-filter reads were aligned to the human reference genome, and a total of 933 million mapped subread bases were generated. Nearly 73% of the aligned subreads mapped to the 7 Mb target region. With an N50 subread length of 4.2 Kb (*i.e.* 50% of the bases are contained in subread lengths longer than 4.2 Kb), the mean mapped subread length was ~2.4 Kb. Slightly lower capture efficiencies were observed for the 1 Kb insert (65%) and 4 Kb insert (69%) SMS/PTLS BAB1123 capture libraries (Table 2; Additional file 3: Table S1), suggesting that longer read lengths potentially improve target specificity in complex genomic regions. The mean mapped subread lengths increased from 770 bp (1 Kb insert library) to ~2.2 Kb (4 Kb insert library). For the two 4 Kb insert *MHC* capture libraries (made from HS1011 and HapMap NA12878), approximately 50% of target specificity was achieved, and the mean mapped subread length reached ~2.1 Kb for the HS1011 library and ~1.9 Kb for the NA12878 library (Table 2; Additional file 3: Table S1).

**Table 2:** **Pilot studies of PacBio-LITS libraries**

**Sample ID**	**Ins. size**	**Probe (target size)**	**Loading /Chemistry/ Movie time (mins)**	Total mapped subreads*	Total mapped SRB (Mb)*	Mean mapped SRL (bp)*	Subreads on target (%)*	Avg. Cov. (x)*	≥1x Cov. (%)*	≥20x Cov. (%)*
BAB1123	1 Kb	SMS/PTLS (7 Mb)	Regular/ C2-P2/1 × 90	360,145	277	770	65	20	89	41
BAB1123	4 Kb	SMS/PTLS (7 Mb)	MagBead/ C2-P2/1 × 120	162,600	353	2,169	69	29	90	54
NA12878	4 Kb	MHC (5 Mb)	MagBead/ C2-XL/1 × 120	214,130	409	1,885	50	44	96	70
HS1011	4 Kb	MHC (5 Mb)	MagBead/ C2-XL/1 × 120	329,251	698	2,101	49	73	97	87
NA12878	6 Kb	SMS/PTLS (7 Mb)	MagBead/ C3-P5/1 × 180	395,342	933	2,358	73	91	95	89

*Detailed sequencing metrics showed in Additional file 3: Table S1.; SRB: subread base; SRL: subread length.

### Insight into complex genomic rearrangements (CGRs) in PTLS cases

PTLS is caused by duplication of 17p11.2, ranging in size from ~0.4 Mb to ~14 Mb (physical position ~8 Mb-22 Mb) and involving the dosage sensitive gene *RAI1* (*Retinoic Acid-Inducible 1* [MIM#607642]) [19-23]. It was previously estimated that at least 23% of the DNA sequences in proximal chromosome 17p (centromere to distal CMT1A-REP) are composed of LCRs, many of which are long and share high identity (>97%) with other repeats in the region [24]. The complete sequencing of chromosome 17 revealed that ~13% of the p arm was composed of LCRs/segmental duplications and that 47% was self-chains, in contrast to genome-wide averages of 5.7% and 23%, respectively (GRCh37/hg19) [25,26]. The majority of duplication events that lead to PTLS are recurrent (either common or uncommon) and are mediated by non-allelic homologous recombination involving LCRs. However, non-recurrent complex genomic rearrangement (CGR) events, which refer to chromosomal rearrangements consisting of two or more breakpoint junctions, have been identified in ~20-30% of PTLS patients [19,20]. Several DNA replication-based mechanisms have been proposed for these CGRs [12,23,27-29], and breakpoint sequencing is useful in discerning which mechanism may have been employed. The breakpoint junctional sequences can provide ‘mutational signatures’ enabling inferences about mechanism used to generate the rearrangements. Analysis of PTLS therefore provides both a generally difficult challenge for DNA sequencing and mapping methodologies as well as an ideal scenario to specifically test the PacBio-LITS approach. Furthermore, complete elucidation of the breakpoint sequences can add to the knowledge of the resultant structural haplotypes of these events.

**Figure 3:**
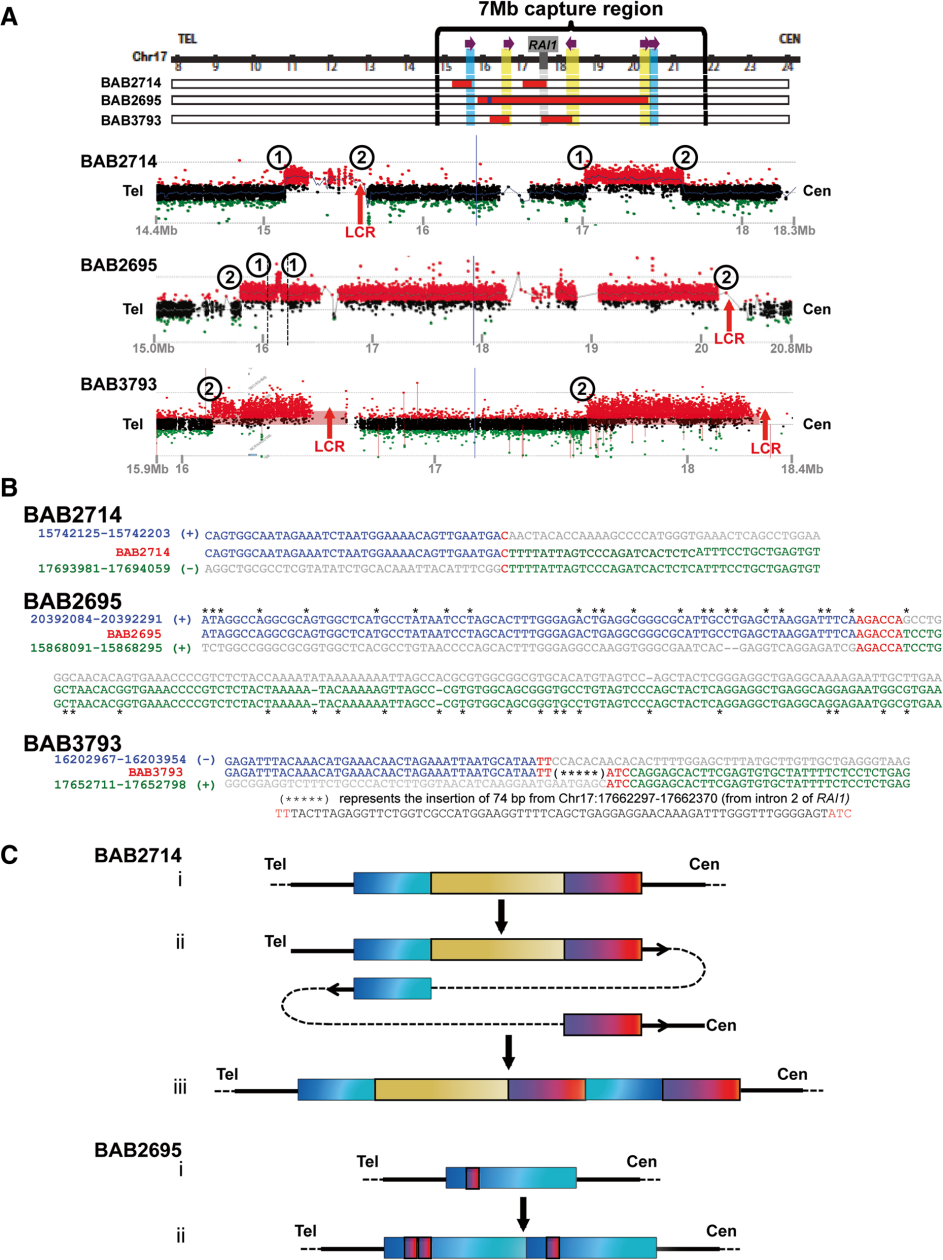
**Delineation of CGRs in PTLS cases. A)** CGRs revealed by aCGH. Human chromosome 17p11.2 is illustrated as a horizontal line on the top of the figure with coordinates (Mb) indicated below. Red blocks represent duplicated regions and blue segments indicate triplications. *RAI1* is indicated by the vertical gray shadow. Yellow and blue shaded areas represent LCRs; purple arrows indicating the orientation [19]. Vertical black lines define the 7 Mb (14.9-21.9 Mb) targeted by the SMS/PTLS probe set. Individual array results are below the schematics, focused on of copy number alterations. Coordinates coordinates (in Mb) are indicated below arrays. Previously determined junctions are labeled with a “1” so that the rearrangement joins together the two number “1”s, and junctions identified by PacBio-LITS are labeled with a “2”. Data for 2714 [19] and 2695 [23] were published previously. **B)** Novel breakpoint junction sequences detected with PacBio-LITS. Breakpoint sequences for the three new junctions identified by PacBio-LITS are aligned to the reference sequence. Transitions between the sequences are indicated with different colors, with gray denoting regions of disagreement with the junction sequence. Chromosome 17 coordinates (hg19/GRCh37) are indicated. Red lettering denotes microhomology. The *Alu-Alu* mediated alignment in BAB2695 has asterisks (*) denoting regions where the two *Alu* elements do not align. **C)** Formation of CGRs. Case BAB2714 i) A map of the reference genome. Colored boxes represent sequence blocks. ii) Black arrows indicate the two template switches resulting in the rearrangement. The template switches could also have occurred in the opposite order. iii) The rearranged region, which has an inversion-duplication for the blue sequence block followed by a direct duplication of the red sequence block. **Case BAB2695** i) A map of the reference genome. ii) The resultant rearrangement. Both junctions are mediated by *Alu* elements, and are in a head-to-tail tandem orientation (no inversion).

Three PTLS cases (BAB2714, BAB2695 and BAB3793) involving CGRs were selected for investigation. The first two cases (BAB2714 and BAB2695) each represented prior partial characterizations of the PTLS region, where previously determined breakpoint junctions may indicate the success of the method. Both of these CGRs harbor four copy number transitions, but previously, only one breakpoint had been elucidated in each patient (Figure 3a, 1 to 1 for BAB2714 and BAB2695) [19,23]. These two previously described breakpoint junctions occurred between *Alu* elements, yielding chimeric *AluY* elements with 33 and 31 bp of microhomology in BAB2714 and BAB2695, respectively [19,23]. However, these CGRs required the sequence of a second breakpoint to fully resolve the SVs (Figure 3a, 2 to 2). Interestingly, the previously undetermined breakpoint junctions in these two patients have one end within an LCR, leading to large uncertainty regions (~62-230 Kb) in the aCGH data, and difficulty mapping the junctions at sequence resolution for breakpoint junctions (Figure 3a). BAB3793 was a new case and not previously published.

We constructed individual 4 Kb insert PacBio-LITS libraries for personal genomes of these three PTLS cases using the custom SMS/PTLS probe set described above. Data analysis revealed that approximately 60-70% of aligned reads mapped to the target region and the mean mapped subread length reached ~1.4-1.9 Kb (Additional file 3: Table S1). Breakpoint analysis using the in-house developed bioinformatic PBHoney tool [30] identified both the known (junction 1) and previously undetermined (junction 2) breakpoint junctions in these three patients (Method; Additional file 4: Table S2), mapping the known breakpoints to within the relevant *Alu* elements [19,23]. Identification of the known variants validated the PacBio-LITS method for CGR breakpoint discovery. Due to the ~15% error rate in single-pass sequences, the precise nucleotide-resolution of the novel junctions required further validation. Designing PCR primers based upon the PacBio results allowed for Sanger confirmation of the junctions and base pair level resolution of the three novel (2 to 2) junctions (Figure 3a, 3b).

The novel breakpoints identified by PacBio-LITS are depicted in Figure 3b. One base pair of microhomology was present at the LCR-mediated inversion rearrangement in BAB2714. The BAB2695 sample contained a second *Alu-Alu* mediated event, resulting in a chimera between an *AluSx* and an *AluY* with 6 bp of microhomology at the junction. In BAB3793, the identified junction represented an inversion, and was a complex event involving two template switches with two and three base pairs of microhomology. The extent of microhomology detected at the junctions in these three PTLS patients suggests replicative mechanisms underlie the formation of the breakpoints [12,23,27-29].

Upon identification of two breakpoint junctions in BAB2714, we propose a mechanism for formation of this structural variant involving a duplication-normal-duplication pattern (Figure 3c). Two template switches likely occurred during the CGR, resulting in an inversion of one of the duplicated segments (blue block) and a dup-nml-dup/inv rearrangement, similar to rearrangements previously observed in Pelizaeus–Merzbacher disease (PMD, MIM#312080) [31]. In BAB2695, the smaller triplicated segment is in tandem [23]. The breakpoint identified by PacBio-LITS in Figure 3b suggests a similar tandem arrangement for the larger duplicated segment, resulting in a dup-trip-dup structure with an internal triplication on one of the duplicated segments (Figure 3c). The rearrangement present in BAB3793 needs further investigation to elucidate the breakpoint within the two LCRs, but given the novel inversion breakpoint depicted in Figure 3b, the likely structural haplotype for this individual is an inversion followed by a tandem duplication, as was previously proposed for BAB2543 [23]. This suggests that there are at least two mechanisms for the formation of dup-nml-dup/inv structures.

## Conclusions

Chromosomal SVs are often difficult to interrogate using microarrays or short-read NGS-based methods. When the rearranged fragments are in regions with complex architecture, such as when flanked by LCRs or other repetitive elements, the challenges are even greater. Other regions of the genome prone to genomic rearrangements exhibit a similar LCR and repetitive DNA makeup as chromosome 17p [32]. Such sequence architecture renders regions of the human genome susceptible to genomic instability [26,32,33].

In this report, we demonstrated a new method, PacBio-LITS, that is an effective approach for elucidation of LCR-mediated breakpoint junctions. The method allows analysis of larger DNA fragments via long sequencing read lengths, than comparable NGS approaches, but does not demand expensive WGS coverage. The method is therefore suitable for studying complex SVs, especially those involving LCRs, inversions, and the generation of chimeric *Alu* elements at the breakpoints.

We demonstrated the efficacy of PacBio-LITS for studying breakpoints in three PTLS samples and identified microhomologies present at the junctions in all three (Figure 3b). Interestingly, when all of the junctions are examined, 3 of the 5 breakpoints resulted in *Alu-Alu* chimeras, which may be difficult to detect with shorter reads [34]. Additionally, in a cohort of 123 PTLS patients ~60% of the non-recurrent rearrangements have one or more breakpoint within an LCR [20]. Therefore, this method will likely be highly useful for determining LCR-mediated breakpoints in genomic disorders.

PacBio-LITS could be extended from studying CGRs to other genomic research applications, such as haplotype phasing and insertion and deletion validation. While conventional long-range PCR amplicon sequencing methods generally render higher specificity (provided that primer design is unique), such methods work best on limited numbers of small-size targets. Optimization of PCR primer designs and reaction conditions is often needed for different targets to overcome unfavorable amplicon sizes and local genome architecture (*e.g.* repetitive sequences, extreme GC contents). The new PacBio-LITS method captures sequences with high-density tiling oligonucleotide probes, thus representing a more robust and efficient approach for targeted structural variation investigations.

So far we have been able to routinely prepare PacBio-LITS libraries with insert sizes up to 8 Kb. With continuous advances in sequencing technology (*e.g.* improvement of read length and error rate), it is possible in the foreseeable future that libraries with even larger inserts will be much desired for SV studies. This would require further optimization of the current experimental conditions, including the likely requirement for more high-quality genomic DNA. To ameliorate PCR amplification issues caused by compromised sample quality (*e.g.* nicks, abasic sites etc.), a DNA repair procedure using commercial kits (*e.g*. PreCR Repair Mix by New England Biolabs) could be incorporated into the protocol. Conversely, new library preparation techniques such as transposon-mediated tagmentation could also be used to reduce input DNA amount and increase ligation efficiency for better PCR performance [35]. To overcome size limitations posed by conventional liquid based capture and PCR amplification, alternative approaches such as restriction enzyme digestion [36] could be explored. Further development of the PacBio-LITS method will allow implementation in a high-throughput fashion for large-scale, genome-wide studies. Although size selection for large insert fragments and PCR validation of SVs may remain labor intensive, migrating from a manual to an automated protocol for other library construction procedures could be achieved by using robotic platforms.

## Methods

### Control and patient samples

Lymphoblastoid cell line-derived HapMap NA12878 DNA and in-house HS1011 blood DNA were utilized as control DNA in the pilot studies. Individuals with PTLS or duplications of 17p11.2 including *RAI1* were obtained by physician referral or self-referral. Patients were enrolled through informed consent under protocol H-9170; control individual HS1011 was enrolled through protocol H-29697. All protocols are approved by the Institutional Review Board at Baylor College of Medicine. These protocols provide informed consent to publish the detailed genomic information contained in this manuscript. Some of the data presented in this manuscript for BAB2714 and BAB2695 appeared previously, as stated in the results section [19,23].

### Library construction, sequencing and probe sets

A detailed protocol for 6 Kb insert capture library preparation is included in Additional file 1. For 1 Kb, 4 Kb and 8 Kb insert capture library preparation, parameters for adjusting the protocol are given in Table 1. Sequencing of the final PacBio capture libraries was conducted by following the manufacturer’s manual guide [37]. For target enrichment, two regional capture probe sets were used in the test experiments. The SMS/PTLS probe set was custom-designed and manufactured by Roche/NimbleGen. It targets a 7 Mb candidate region on chr.17p11.2 for reciprocal genomic disorders SMS and PTLS. The human MHC probe set (*Design Name: 110729_HG19_MHC_L2R_D03_EZ*) was kindly provided by Roche/NimbleGen and targets a 4.97 Mb human MHC region including 8 known haplotypes.

### Data analysis

Filtered subreads were generated using SMRTAnalysis (provided by Pacific Biosciences, MenloPark CA) and mapped to the Human reference genome GRCh37 (hg19) using PacBio long read aligner BLASR [38], an alignment tool optimized for reads thousands of base pairs long with higher error rates. The following BLASR parameters were set: −affineAlign -noSplitSubreads -nCandidates 20 -minPctIdentity 75 -sdpTupleSize 6 -minLength 100. The resulting BAM alignment files were processed to generate capture metrics using the bed files of the tested probe sets (*i.e.* SMS/PTLS and MHC).

For analysis of BAB2714, BAB2695, and BAB3793, PBHoney-Tails [30] (https://www.hgsc.bcm.edu/software/honey) was run with the following parameters: −buffer 1000 -minBreads 25 -minZMWs 25 -minMapq 150. Briefly, after an initial mapping with BLASR, soft-clipped (*i.e.* unmapped) tails were extracted from the SAM alignment output file and remapped to the hg19 reference genome with BLASR. The initial alignment reads and their re-mapped tails composed “piece-alignments”, which were then clustered if similarly mapped tails were shared based on location and orientation. Buffer distance was set at 1000 bp in this work, and clusters with a minimum of 25 piece-alignments and a minimum average Phred-scale mapping quality value of 150 were subjected to further examination for detection of candidate breakpoints. These minimum threshold parameters helped exclude piece-alignments that were possibly the result of chimeras associated with library preparation and sequencing.

The total numbers of putative breakpoint junctions discovered by PBHoney for BAB2714, BAB2695, and BAB3793 were 13, 16, and 15, respectively (Additional file 4: Table S2). We subsequently screened the putative results for any candidate junction with breakpoints falling in the SMS/PTLS probe target region and intersecting with the aCGH results. With the above filtering parameters, two breakpoint junctions within the aCGH predicted regions (one previously reported [19,23] and one novel) were identified for each of BAB2714 and BAB2695, while BAB3793 only had one breakpoint junction called. Validation of the novel variants was conducted and base pair level resolution of breakpoints was achieved by performing Sanger sequencing.

## Additional files

Additional file 1:
**BCM-HGSC PacBio-LITS Protocol.** Preparation of 6 Kb insert capture libraries for PacBio long-read length sequencing. Additional file 2: Figure S1. Selecting the correct DNA polymerase for PCR amplification. Additional file 3: Table S1. Summary of capture and sequencing metrics from the PacBio-LITS studies. Additional file 4: Table S2. Summary of breakpoint analysis for the three PTLS cases.

## Abbreviations

WGS: Whole genome sequencing
SV: Structural variation
LCR: Low copy repeat
PTLS: Potocki–Lupski syndrome
HapMap: Human haplotype map
aCGH: Array comparative genomic hybridization
NGS: Next-generation sequencing
LM-PCR: Ligation-mediated PCR
SMS: Smith-Magenis syndrome
MHC: Major histocompatibility complex
CGR: Complex genomic rearrangement.
