# Supplementary material for: PacBio-LITS: a large-insert targeted sequencing method for characterization of human disease-associated chromosomal structural variations
Source: BMC Genomics. 2015 Mar 19;16(1):214. doi: 10.1186/s12864-015-1370-2 (PMC4376517; doi:10.1186/s12864-015-1370-2)
Supplement: Additional file 1: — BCM-HGSC PacBio-LITS Protocol. Preparation of 6 Kb insert capture libraries for PacBio long-read length sequencing. [file 12864_2015_1370_MOESM1_ESM.doc]

**Additional file 1: BCM-HGSC PacBio-LITS Protocol**

**Preparation of 6 Kb insert NimbleGen capture libraries for PacBio long-read length sequencing**

This protocol provides the instruction for preparing large-insert (6 Kb) target enrichment libraries for PacBio long-read length sequencing. The entire process involves 1) DNA fragmentation and size selection; 2) pre-capture library preparation; 3) target enrichment with NimbleGen manufactured probes and 4) post-capture PacBio library preparation**. It is highly recommended that first-time users start with smaller insert sizes (i.e. 1 kb and 4 kb) using conditions described in Table 1 of the main text to practice procedures and test instruments and reagents.**

References:

1. Covaris g-TUBE User Manual Part number 010154 Rev C.

2. [Agilent DNA 7500 and DNA 12000 Kit Guide](http://www.chem.agilent.com/library/usermanuals/Public/G2938-90024_DNA7500-12000_KG.pdf) Part number: G2938-90024 (Rev. B)

3. [Agilent High Sensitivity DNA Kit Guide](http://www.chem.agilent.com/library/usermanuals/Public/G2938-90321_SensitivityDNA_KG_EN.pdf) Part number G2938-90321 Rev. B

4. Sage Science Blue Pippin DNA Size Selection System Operations Manual Software v.6.00, cassette definition set 10.

5. [NimbleGen SeqCap EZ Library SR User's Guide v4.1](https://www.google.com/url?sa=t&rct=j&q=&esrc=s&source=web&cd=1&cad=rja&ved=0CCcQFjAA&url=https://www.roche-applied-science.com/wcsstore/RASCatalogAssetStore/Articles/SeqCapEZLibrarySR_UGuide_v4p1.pdf&ei=JGX8UsSyJbHlyAGui4HoCA&usg=AFQjCNGjMVRz5v-POqnsEvn37GfsFZAj9A)

6. PacBio Procedure & Checklist: 10 Kb Template Preparation and Sequencing (With Low-Input DNA) Part no.100-152-400-04.

**Step 1. DNA fragmentation and size selection**

- 1. Shear the DNA into 6 Kb fragment using Covaris g-TUBE (C**at.no.** 520079) under the following condition:

Mass of DNA: 1 μg

Buffer: 1xTE

Shearing volume: 150 μl

Eppendorf 5424 centrifuge speed (RPM): 7000 rpm

Processing time (minutes): 3

1.2 Clean up/concentrate the sheared DNA with 0.8x SPRI AMPure XP beads.

A) add 120μl SPRI AMPure XP beads into each tubes.

B) mix the DNA/Beads on thermomixer in the highest speed (1400rpm) for 5 minutes at RT.

C) place the tubes into the DynaMag™-2 magnet device for 2 minutes until the solution clears. Remove and discard the supernatant.

D) wash two times with freshly prepared 70% ethanol. Keep the sample tubes in the DynaMag™-2 magnet device during the washing and avoid disturbing the bead pellet. After washing, leave the tube in the DynaMag™-2 magnet device for 2 minutes at RT, then elute DNA in 32 µl nuclease-free H2O.

1.3 **QC:** Run 1 μl of the sheared DNA on Agilent 2100 Bioanalyzer using DNA 12000 Chip (Cat. no. 5067-1508) to check size distribution and quantity (see example below).


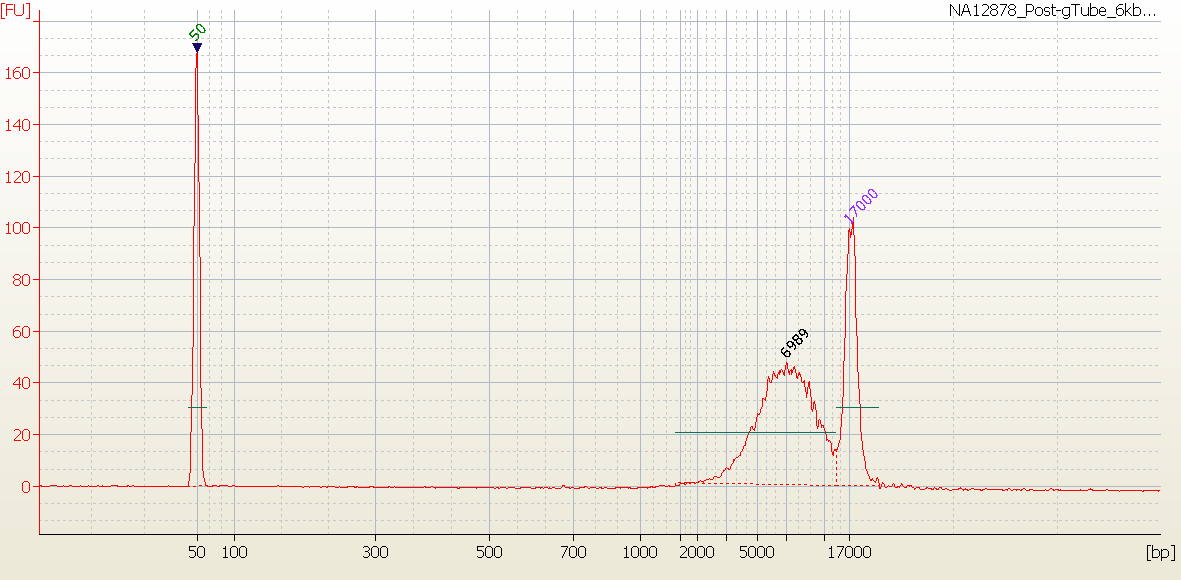


**Figure 1 Post g-TUBE shearing QC of a PacBio 6Kb insert capture library on Agilent 2100 Bioanalyzer (DNA 12000 chip).**

- 1. Size selection of the sheared DNA using BluePippin

A) Combine 30μl of the eluted DNA with 10μl of loading solution in a 1.7μl Eppendorf tube. Briefly vortex to mix the sample solution and quickly spin down.

B) Load the sample solution to BluePippin 0.75% agarose gel cassette (**Product number BLF 7510**) and run size selection with the below setting:

**Cassette definition: 0.75% DF 3-10Kb Marker S1**

**Size selection range: 5000bp-9000bp**

C) After the run ends, collect the sample DNA from the elution module using a standard 100-200μl pipette. To improve DNA recovery, wash the elution module with 40μl 0.1%Tween 20. Remove the buffer-Tween solution and combine it with the initial DNA elution product.

D) Clean up the size-selected product with 0.8x SPRI AMPure XP beads. Elute DNA to 78μl.

**Note: Size selection step could also be performed after adaptor ligation.**

1.5 **QC**: Run 1 μl of purified DNA on Agilent 2100 Bioanalyzer using DNA High Sensitivity Chip (Cat no. 5067-4626) to check size distribution and quantity (see example below).


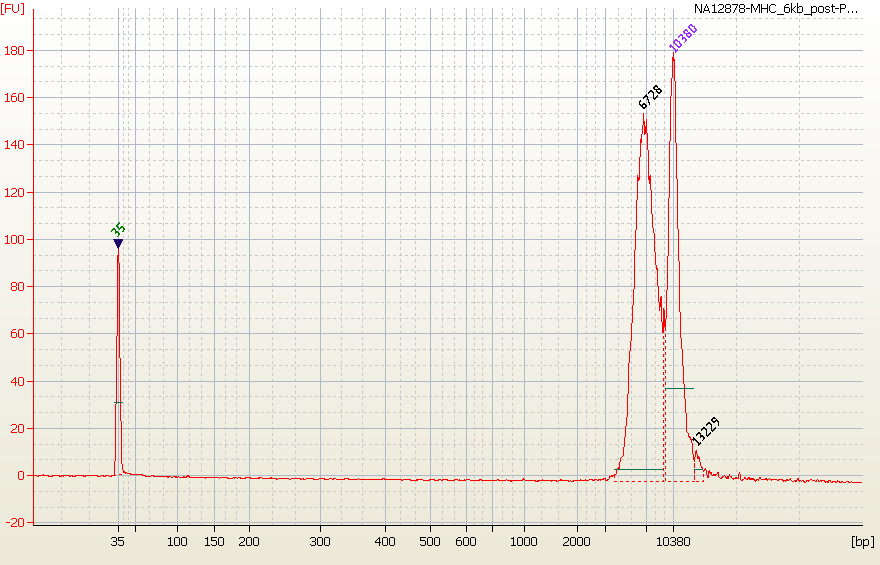


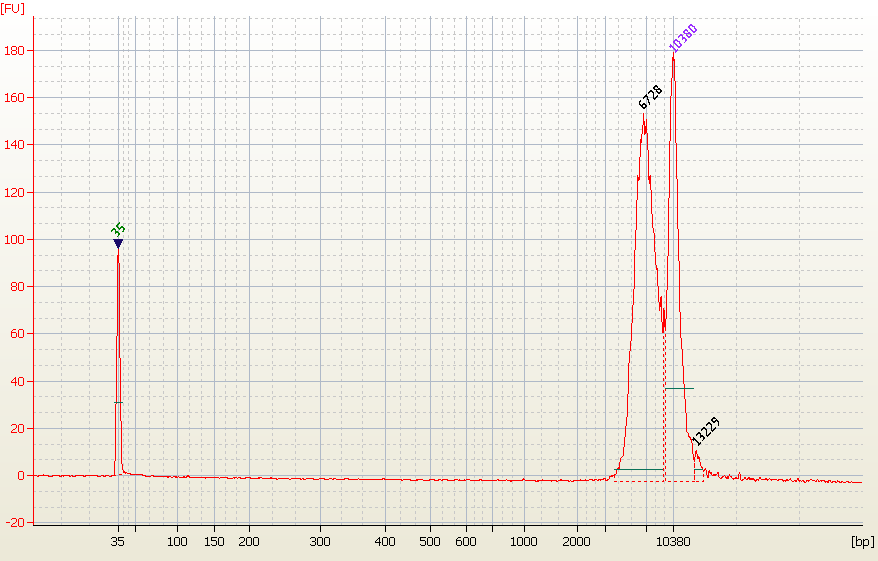


**Figure 2 Post BluePippin size selection QC of a PacBio 6Kb insert capture library on Agilent 2100 Bioanalyzer (High sensitivity chip).**

**Step 2. Pre-capture library preparation**

**2.1 DNA end-repair**

A) Combine and mix the following components into the sample tubes:

| **Component** | **Volume (μL)** |
| --- | --- |
| Size selected DNA | 76.0 |
| End-repair 10× buffer* | 9.0 |
| End-repair enzyme mix* | 5.0 |
| Total | 90.0 |

*From NEBNext End-Repair Module (Cat. No. E6050L).

B) Incubate the mixture at 25°C for 30 minutes at a bench top thermomixer.

C) Purify with 0.8x SPRI AMPure XP beads and elute the DNA sample in 52 µl nuclease-free H2O.

**2.2 3’-end adenylation**

A) Combine and mix the following components in the sample tubes:

| **Component** | **Volume (μL)** |
| --- | --- |
| End-repaired DNA | 51.0 |
| NEBNext^TM^ dA-Tailing Reaction Buffer (10X)* | 6.0 |
| Klenow Fragment (3’-5’ exo^-^)* | 3.0 |
| Total | 60.0 |

*From NEBNext dA-Tailing Module (Cat. No. E6053L).

B) Incubate the mixture at 37**°**C thermomixer for 20 min.

C) Purify with 0.8X SPRI AMPure XP beads and elute the DNA sample in 64μl nuclease-free H2O.

**2.3 Ligation of Illumina index paired-end adaptors**

A) Combine and mix the components in the sample tubes:

| **Component** | **Volume (μL)** |
| --- | --- |
| Illumina Index Paired-end Adaptor (15 μM) | 5.0 |
| Quick Ligase 5X buffer* | 18.0 |
| A-Tailed DNA | 62.0 |
| Quick Ligase Enzyme* | 5.0 |
| Total | 90.0 |

* From NEB (Cat. No. E-6056L).

B) Incubate at room temperature for 30 minutes.

C) Purify with 0.8x SPRI AMPure XP beads and elute DNA in 72µl nuclease-free H2O. Transfer the eluted ligation sample (~70µl in volume) into a new 0.2ml PCR strip tube.

**2.4 Pre-capture ligation-mediated PCR (LM-PCR)**

A) Add and mix the following PCR reagents to the PCR strip tube

| **Component** | **Volume (μL)** |
| --- | --- |
| *10x LA PCR Buffer | 10.0 |
| dNTP (2.5mM) | 16.0 |
| LM-PCR primer 1.0 (50µM) | 2.0 |
| LM-PCR primer 2.0 (50µM) | 2.0 |
| *TaKaRa LA *Taq* DNA polymerase (Hot Start) | 0.6 |
| Total reaction volume | 100.0 |

* Clontech, Cat no. RR042.

B) Seal the tube with cap. Place it in the ABI GeneAmp PCR System 9700 for PCR amplification under the following PCR condition:

(1) 2’ @ 95ºC

(2) 20” @ 95ºC

(3) 10’ @ 68ºC

Repeat step (2) to (3) for total 10-12 cycles

(4) 10’ @ 72ºC

(5) HOLD @ 4ºC

(6) END

C) After PCR, load 1 μl of PCR product on a 1.2% FlashGel and run the product along with FlashGel DNA ladder. **Avoid over-amplification.**

D) If PCR product appears clean on the gel, clean up the PCR product using 0.8x SPRI AMPure XP beads and elute it in 42µl nuclease- free H2O. **Avoid vigorously pipetting or vortexing during the purification.** (Option: a second round of 0.6x SPRI AMPure beads purification could be performed to improve target capture performance).

**2.5 QC**: Run 1 μl of purified DNA on Agilent 2100 Bioanalyzer using using DNA 12000 Chip (cat. no. 5067-1508) to check size distribution and quantity (see example below). **It is critical that the amplified product exhibits clean peak on Agilent Bioanalyzer. Small size of fragments in the pre-capture library will be over-amplified in post-capture PCR amplification thus decreasing the final capture library size.**


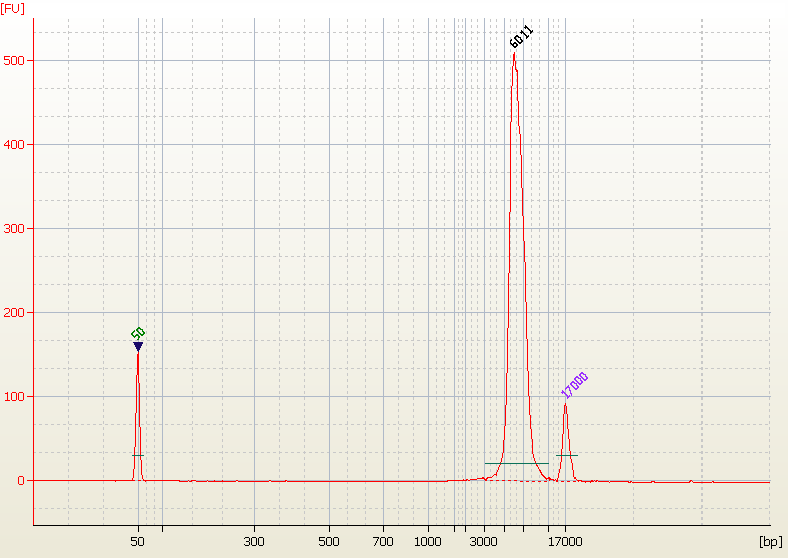

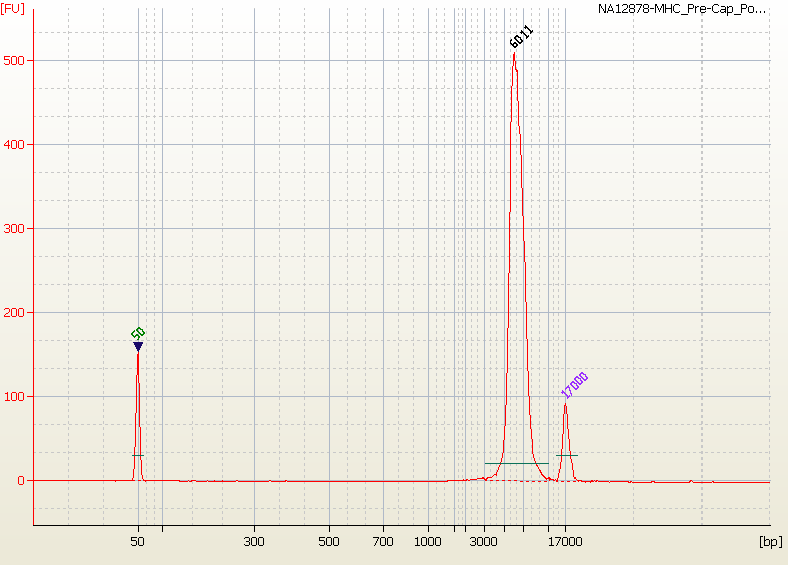


**Figure 3 Pre-capture library QC of a PacBio 6Kb insert capture library on Agilent 2100 Bioanalyzer (DNA 12000 chip).**

**Step 3.Target enrichment with NimbleGen probes**

Follow NimbleGen solution-based sequence capture protocol (reference 5) to conduct target enrichment. **It is critical to avoid vigorously pipetting and vortexing during the entire target enrichment process.** Use 1-2µg DNA in each hybridization reaction. A 36-48 hour incubation at 47°C is generally sufficient for probe-DNA binding. Although Illumina index adaptors enable multiplex co-capture, single sample capture is currently recommended due to the challenge associated with de-multiplexing large-insert PacBio pools (see the Result and Discussion section). Perform post-capture LM-PCR for 14-18 cycles under the same cycling conditions used in the pre-capture LM-PCR. **Avoid over-amplification.**

**Step 4. Post-capture PacBio library preparation.**

Follow PacBio library preparation protocol (reference 6) to add SMRT bell adaptors to the capture product. **Do not perform DNA shearing**. 500ng-1µg post-capture library DNA is generally needed. The final PacBio library should be clean and show a single sharp peak on Agilent 2100 Bioanalyzer. Over-amplification in pre- and post-capture PCR steps could generate small-fragments in the final library, leading to sub-optimal sequencing performance. A second round of size selection may be performed to remove the small fragments.

**Appendix:**

**Illumina index adaptor oligos (*addition of phosphothioate bond before addition of the last “T”)**

| Illu-A-ID 1 | /5Phos/GAT CGG AAG AGC ACA CGT CTG AAC TCC AGT CAC ATC ACG ATC TCG TAT GCC GTC TTC TGC TTG |
| --- | --- |
| Illu-A-ID 2 | /5Phos/GAT CGG AAG AGC ACA CGT CTG AAC TCC AGT CAC CGA TGT ATC TCG TAT GCC GTC TTC TGC TTG |
| Illu-A-ID 3 | /5Phos/GAT CGG AAG AGC ACA CGT CTG AAC TCC AGT CAC TTA GGC ATC TCG TAT GCC GTC TTC TGC TTG |
| Illu-A-ID 4 | /5Phos/GAT CGG AAG AGC ACA CGT CTG AAC TCC AGT CAC TGA CCA ATC TCG TAT GCC GTC TTC TGC TTG |
| Illu-A-ID 5 | /5Phos/GAT CGG AAG AGC ACA CGT CTG AAC TCC AGT CAC ACA GTG ATC TCG TAT GCC GTC TTC TGC TTG |
| Illu-A-ID 6 | /5Phos/GAT CGG AAG AGC ACA CGT CTG AAC TCC AGT CAC GCC AAT ATC TCG TAT GCC GTC TTC TGC TTG |
| Illu-A-ID 7 | /5Phos/GAT CGG AAG AGC ACA CGT CTG AAC TCC AGT CAC CAG ATC ATC TCG TAT GCC GTC TTC TGC TTG |
| Illu-A-ID 8 | /5Phos/GAT CGG AAG AGC ACA CGT CTG AAC TCC AGT CAC ACT TGA ATC TCG TAT GCC GTC TTC TGC TTG |
| Illu-A-ID 9 | /5Phos/GAT CGG AAG AGC ACA CGT CTG AAC TCC AGT CAC GAT CAG ATC TCG TAT GCC GTC TTC TGC TTG |
| Illu-A-ID 10 | /5Phos/GAT CGG AAG AGC ACA CGT CTG AAC TCC AGT CAC TAG CTT ATC TCG TAT GCC GTC TTC TGC TTG |
| Illu-A-ID 11 | /5Phos/GAT CGG AAG AGC ACA CGT CTG AAC TCC AGT CAC GGC TAC ATC TCG TAT GCC GTC TTC TGC TTG |
| Illu-A-ID 12 | /5Phos/GAT CGG AAG AGC ACA CGT CTG AAC TCC AGT CAC CTT GTA ATC TCG TAT GCC GTC TTC TGC TTG |
| Illu-A-universal | 5’-AAT GAT ACG GCG ACC ACC GAG ATC TAC ACT CTT TCC CTA CAC GAC GCT CTT CCG ATC* T |

**Note: Illu-A-IDn (n=1-12) and Illu-A-universal oligos should be pre-annealed using the following protocol:**

Mix the paired oligonuclieotides at final concentration of 300uM in 1x Ligase buffer (from NEBNext Ligation Module, Cat# E6056-L) and run with the following annealing program on thermocycler: 95°C for 5 min, 80°C for 3 min, 70°C for 3 min, 60°C for 3 min, 50°C for 3 min, 40°C for 3 min, 30°C for 3 min, 20°C for 3 min and 4°C hold. Dilute 20x to obtain 15uM working concentration. Aliquot annealed adaptor into eppendorf tubes and store them in -20°C freezer. Record the date.

**LM-PCR primer 1.0**

5’-AATGATACGGCGACCACCGAGA

**LM-PCR primer 2.0**

5’-CAAGCAGAAGACGGCATACGAG

**(end)**
