# Supplementary material for: PacBio-LITS: a large-insert targeted sequencing method for characterization of human disease-associated chromosomal structural variations
Source: BMC Genomics. 2015 Mar 19;16(1):214. doi: 10.1186/s12864-015-1370-2 (PMC4376517; doi:10.1186/s12864-015-1370-2)

**Additional file 2: Figure S1.** Selecting the correct DNA polymerase for PCR amplification. Robust generation of clean PCR products in LM-PCR is critical to the successful large insert capture library preparation. Here, two different DNA polymerases were evaluated for the 6 Kb library construction. TaKaRa LA *Taq* Polymerase (Cat. # RR042, Clontech Lab. Inc.) generates more robust amplification than Phusion HiFi DNA polymerase (Cat. # M0530, NEB Inc). The PCR products were run on a 1.2% FlashGel DNA Cassette (Cat. # 57023, Lonza Group Ltd.) along with the 100bp-4Kb FlashGel DNA Marker (Cat. # 50473, Lonza Group Ltd.).


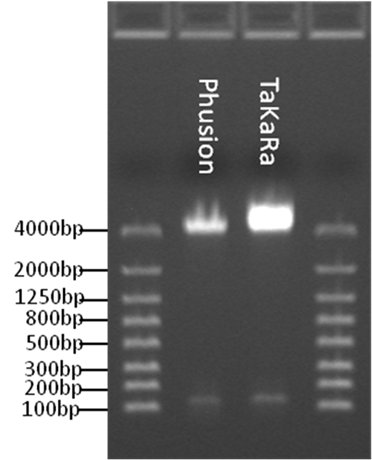

Supplement: Additional file 2: Figure S1. — Selecting the correct DNA polymerase for PCR amplification. [file 12864_2015_1370_MOESM2_ESM.doc]
